# Wisdom from the past

**Published:** 1970-01

**Authors:** 


### Wisdom from the Past

**Extracts From This Journal — 1894 and 1918**

### SEVENTY-SIX YEARS AGO

Extract from a paper on " Intussusception " by Arthur W. Pritchard, M.R.C.S., Eng., Senior Surgeon to the Bristol Royal Infirmary; Lecturer on Practical Surgery in University College, Bristol.

"With regard to the cause : to me it seems wonderful, when we think how active and apparently partial and irregular peristalsis is, that intussusception does not more commonly occur. Anything that interferes with the proper regulation of the rhythmical movement of the intestine may cause it, and increased excitement in one part from the presence of an irritating substance—such as a piece of undigested food—probably commonly gives rise to it. Occasionally a polypus or other tumour of the intestinal wall has been shown to have excited it. Decreased or disordered intestinal movements from want of proper nerve-control, such as may occur in certain cerebral lesions, may predispose to it; and under this head we must place that curious phenomenon of intussusception of the dying, which is not uncommon. There is on the table a very good specimen of this condition, showing multiple intussuscepta, easily reducible, and not all pointing in the same direction, in some places the upper segment of the bowel having slipped inside the lower, and in others the lower inside the upper. But of all causes, I think that, in children at all events, it is very likely that the condition is more or less directly due to external violence. We know that a great proportion of the cases occur in children. It is very common under the age of one year, and more than half the cases occur in those under the age of 10. When we think of the way in which infants are hurriedly snatched up, and carried doubled up with the hand or arm around the abdomen, can we be astonished at a part of the intestine becoming injured and perhaps paralysed by the undue pressure through the thin abdominal wall? Irregular peristalsis thus set up may be the cause of an intussusception, which manifests itself by the child screaming with abdominal pain an hour of two afterwards, the hasty lift being forgotten from constant usage, or perhaps, on the part of the nursemaid, denied. I cannot help saying in this place that I think it quite possible that intussception occurs more frequently than we generally suppose, because of the very ready way in which it may be caused. A great many children suffer from severe abdominal pain that may or may not be followed by vomiting and diarrhœa, symptoms which give way to an opiate. Is it not likely that some of these cases are due to a small intussusception, which rights itself and gives rise to no subsequent trouble? When the pain is referred definitely to the region of the ileo-cæcal valve, the attack being paroxysmal and severe, it is sometimes recognised as being an instance of ileo-cæcal intussception, and a tumour that disappears spontaneously can be felt in that neighbourhood. I am, however, inclined to think that we might find more cases of intussusception if we had opportunities of palpating the abdomen before Nature cured the patient."

(Vol. XII p. 1, 1894)

### Preparations for the Sick

"The 'Riphokam.' — J. Rogerson, London. — Proceeding on the theory that compared with the front part of the body the back is inadequately protected by the ordinary clothing, Mr. Rogerson has devised and patented a double-back under vest, which is well made, and which we can testify from personal experience is exceedingly comfortable to wear. The invention does not consist in adding to the thickness by merely laying one piece of material over another, but by the manner of weaving, the porous character of the garment is still preserved, while the risk of chill is lessened. If any of the obscure neuropathies or pulmonary troubles are caused by insufficient covering of the spinal column or back, this vest ought to be a prophylactic. Mr. Standerwick of Stokes Croft is Mr. Rogerson's local agent."

(Vol. XII p. 267, 1894)

### FIFTY-TWO YEARS AGO
### Hospital Amalgamation

"It has often been said that the chief factor in modern social and political developments has been the immense improvement which has taken place in means of communication and transport. The result of bringing distant parts of the world so close together has been, in all spheres of activity, an enlargement of the effective unit of competition. Towns which were formerly rivals have to combine against distant competitors; large businesses amalgamate; where formerly small states competed with one another international leagues are instituted to carry out wider programmes of rivalry.

"To turn from international to domestic matters, it has become apparent to many both inside and outside the profession that in Bristol there is no longer room for rivalry between the various hospital institutions, and that if the city is to maintain its position as a centre for the advancement and teaching of medical science, it must be ready to compete as a combined unit with the other large cities of the kingdom, and of the empire. These ideas have begun to take practical form. A committee consisting of representatives of the governing bodies and professional staffs of the leading hospitals of the city has been formed and is considering a scheme for amalgamation of both the administrative and medical work of these institutions. Additional stimulus has been given to the movement by the action of the Board of Education, which is prepared to assist medical education and research in a very substantial way by means of grants from national revenue. To obtain the full benefit of this scheme it is, in the opinion of the medical staffs, essential that their resources should be combined; and this, we understand, is also the view taken by the Board of Education. From the financial and administrative point of view there are obvious advantages in amalgamation, as it will avoid much duplication of apparatus and overlapping of interests, while it will enable all purchases to be made in larger quantity and at a cheaper rate. There are, of course, difficulties in detail, but none are insuperable, and it is to be hoped and expected that before long Bristol will possess a centralised and amalgamated system of medical institutions which will enable it to more than hold its own among the great centres of medical activity in the kingdom."

(Vol. XXXVI p. 92, 1918)

(The Bristol Royal Hospital was eventually formed in 1940.—Ed.)

